# The Clinical Pharmacist-Led Consultation for Infectious Diseases in Guizhou Province, China: A Survey Among Hospital Pharmacies

**DOI:** 10.3389/fphar.2020.00149

**Published:** 2020-02-27

**Authors:** Jiaxing Zhang, Chang Xu, Wenyi Zheng, Rui He, Juan Xie, Xin Qian, Shijuan Xiong, Qi Chen

**Affiliations:** ^1^ Department of Pharmacy, Guizhou Provincial People’s Hospital, Guiyang, China; ^2^ Department of Population Medicine, College of Medicine, Qatar University, Doha, Qatar; ^3^ Experimental Cancer Medicine, Clinical Research Center, Department of Laboratory Medicine, Karolinska Institute, Stockholm, Sweden

**Keywords:** infectious diseases, antimicrobial resistance, clinical pharmacist-led consultation, hospital pharmacy, survey

## Abstract

**Background:**

As antimicrobial resistance became an increasing threat to global public health, Clinical Pharmacist-led Consultation (CPC) for Infectious Diseases (ID) was developed in China. We conducted a survey among hospital pharmacies to investigate the present situation and potential determinants of CPC for ID in China's Guizhou Province.

**Methods:**

The study was conducted by sending the questionnaire to the directors of hospital pharmacy who were members of Guizhou Province Pharmaceutical Administration Collaboration Network (GPPACN) from April to May 2019. We implemented the Firth's logistic regression model to analyze the factors that influence the implementation of CPC. The acceptance rate of consultation suggestions (ARCS) and effective response rate after acceptance of pharmacists' suggestions (ERRAPS) were pooled by meta-analysis using a random effect model, respectively. A pairwise meta-analysis was performed to compare the effective response rate between patients whose treatment followed the pharmacists' suggestions and those whose treatment did not.

**Results:**

A total of 83 hospitals covering 9 regions in Guizhou Province were included in this survey. The results showed that 50 hospitals (60.24%) developed the CPC for ID. Staffing ID, respiratory, or general clinical pharmacist had a significantly positive effect on the implementation of CPC [adjusted odds ratios = 7.298, 95% *CI* (confidence interval): 2.232 to 23.858]. The pooled ARCS and ERRAPS for secondary hospitals were 97.59% (95% *CI*: 94.27 to 100.00%) and 88.36% (95% *CI*: 84.71 to 92.17%), respectively. Importantly, CPC improved the prognosis of ID patients [risk ratio (*RR*) = 6.49, 95% *CI*: 2.84 to 14.82] in these hospitals.

**Conclusion:**

CPC adoption is gradually increasing among hospitals and will be a promising intervention for ID treatment at grassroots medical institutions in Guizhou Province. Training more clinical pharmacists to specialize in ID, respiratory, or general diseases might be the most practical measure to promote the development of CPC for ID.

## Introduction

Multidrug-resistant (MDR) infections are an increasing threat to global public health (Marston et al., 2016). At present, the mortality of resistant infections is approximate 700,000 persons per year globally, with a prediction of 10 million people per year by 2050 if no steps are taken to address this issue (O'Neill, 2016; Thakur and Gray, 2019). Many countries have initiated Antimicrobial Stewardship Program (ASP) to combat MDR since the World Health Organization (WHO) called for “no action today, no cure tomorrow” in 2011 (Charani et al., 2019). Clinical Pharmacist-led Consultation (CPC) service, as one part of ASP, was gradually developed in China. Due to an insufficient number of clinical pharmacists, CPC become the main mode of clinical pharmacists' intervention in Chinese hospitals, both in the management of antimicrobials and in the treatment of infectious diseases (ID). A previous systematic review indicated that, in China, CPC had an excellent acceptance of clinicians and improved the prognosis of ID patients [risk ratio (*RR*): 2.08, 95% confidence interval (*CI*): 1.41 to 3.06] (Zhang et al., 2019a). Another prospective cohort study also proved the significant role of CPC on ID treatment [adjusted odds ratio (*AOR*): 1.738, 95% *CI*: 1.028 to 2.940] (Zhang et al., 2019b).

According to the subgroup analyses results of a systematic review (Zhang et al., 2019a), the acceptance rate of consultation suggestion (ARCS) for hospitals in Western China was much higher than that in other developed regions, indicating that clinical pharmacists took an active role in the less developed regions. Guizhou is a developing province in the west of China, with a gross domestic product (GDP) of 209,163.15 million dollars (ranked 23^rd^ among the 31 provinces in China) in 2018. In addition, the number of pharmacists in Guizhou Province was only 8,128 (ranked 23^rd^ among the 31 provinces), according to the 2017 Chinese Health Statistics Yearbook (National Health and Family Planning Commission of the People's Republic of China). A report on the health index published in 2015 pointed out that Guizhou Province was at the bottom of the list and far from the target (19.3601 *vs*. 39.9756) (Bao, 2016). As Guizhou Province is representative of the developing regions in China, its experiences in implementing CPC for ID may be shared with other similar regions or countries.

A survey in 2016 indicated that the status of CPC for ID in Guizhou Province was in an early development stage (Zhang et al., 2018). Unfortunately, due to the absence of follow-up data, that survey could not further evaluate the effectiveness of CPC in treatment of ID. Moreover, during the past 2 years, policy-makers from Guizhou Province have taken a series of measures to promote CPC for ID, including training clinical pharmacists, conducting continuing education courses, establishing telemedicine platforms, and allocating senior clinical pharmacists to the grassroots medical institutions. This present survey, as an extension of the 2016 survey, was thus conducted to fully investigate the current situation and potential determinants of CPC for ID in Guizhou Province.

## Materials and Methods

### Study Design and Sample

The study was a survey among hospital pharmacies in Guizhou Province in China, and the data was collected from the secondary and tertiary hospitals whose pharmacies were affiliated with the Quality Control Center of Guizhou Province Pharmaceutical Administration. The study was approved by the Ethics Committee of Guizhou Provincial People’s Hospital (2017066, **Supplementary Tables S1** and **S2**). Written informed consent for participation was not required for this study in accordance with the national legislation and the institutional requirements.

### Study Measures

A questionnaire, whose reliability and validity had been verified in the previous study (Zhang et al., 2018), was used to investigate the CPC service for ID in Guizhou Province and explore important factors associated with the implementation. The questionnaire contained 13 items that addressed: a) contact information of the director of hospital pharmacy who completed the questionnaire; b) information of the hospital: name, rank, and geographical location; c) situation of clinical pharmacy service (CPS) and clinical pharmacists in the hospital: whether the hospital developed CPS, whether the hospital staffed clinical pharmacists, number, and specialty of the clinical pharmacist; d) situation of CPC for ID in hospital: whether the hospital developed CPC for ID; number of patients who applied for CPC for ID in 2018, number of patients whose treatment followed pharmacists' suggestions in 2018, and number of responsive patients whose treatment followed or did not follow pharmacists' suggestions in 2018.

The ARCS from clinical pharmacists was identified by comparing the consistence between clinicians´ prescriptions and pharmacists´ suggestions. Acceptance indicates that the clinicians completely or partially adopted the advice from pharmacists, and ARCS was presented as the percentage of the acceptance (Zhang et al., 2019a) and calculated using the following formula:

$$\text{ARCS }\left(\%\right)=\;\frac{\text{number of patients whose treatment followed pharmacists' suggestions}}{\text{number of patients who applied for CPC for ID}}\;\times \;100\%$$

Effective response was defined as partial or complete resolution of clinically significant infectious signs/symptoms, improvement or resolution of computed tomography (CT) or magnetic resonance imaging (MRI) findings, and on proven or negative culture results (Zhang et al., 2019b). Response rate was the proportion of patients achieving effective response to total patients. Effective Response Rate after Acceptance of Pharmacists' Suggestions (ERRAPS) was calculated using the following formula:

$$\text{ERRAPS}(\%)=\frac{\text{number of responsive patients whose treatment followed pharmacists}\prime \text{suggestions}}{\text{number of patients whose treatment followed pharmacisist}\prime \text{suggestions}}\times 100\%$$

### Data Collection

Information was collected from the directors of hospital pharmacy who were the members of Guizhou Province Pharmaceutical Administration Collaboration Network (GPPACN). The directors completed the questionnaire according to the registration information of medical institutions and the working record of the pharmacy. Questionnaire Star (Changsha Ranxing Information Technology Co., Ltd), a professional online questionnaire software platform, was used to design and create a link to the questionnaire. The link was then sent *via* WeChat (Shenzhen City Tencent Computer System Co., Ltd) to the pharmacy directors by GPPACN. The survey was carried out among 83 hospital pharmacies from April to May 2019.

### Statistical Analysis

Statistical analysis was conducted using software Stata 14.0. SE. Rate or constituent ratio was used to describe qualitative data. A logistic regression model was utilized. To explore the factors associated with the implementation of CPC for ID, the implementation of CPC for ID was used as the dependent variable while other factors were used as the independent variables. Considering the potential zero events, we used the Firth's logistic regression to fit implementation of CPC for ID against variables, including hospital rank, region, clinical pharmacist sufficiency, staffing ID, respiratory, or general clinical pharmacist.

If more than one hospital reported the same outcome of ARCS or effective response rate after acceptance of pharmacists' suggestions (ERRAPS), a single-arm meta-analysis was utilized. We achieved an exact binomial 95% *CI* of ARCS and ERRAPS in each hospital. The inter-hospital variance was estimated and the pooled estimates of ARC and ERRAPS was determined by the software R 3.3.1 with a random-effect model (Luo et al., 2013).

If more than one hospital compared the effective response rate between patients whose treatment followed the pharmacists' suggestions and those whose treatment did not, a pairwise meta-analysis was conducted. The pooled risk ratios (RRs) were calculated using RevMan 5.3. Heterogeneity among studies was assessed using Cochrane's Q-statistic and *I^2^* test. We applied a fixed-effects model to synthesize data when heterogeneity was not significant (*P* > 0.1 and *I^2^* < 50%). When heterogeneity was significant (*P* ≤ 0.1 and *I^2^* ≥ 50%) and could not be explained by subgroup analyses or in terms of clinical features, a random-effects model was used. We explored the sources of heterogeneity based on the following subgroup analyses: hospital rank (secondary hospital *vs.* tertiary hospital).

## Results

### Characteristics of Included Hospitals

**Figure 1:**
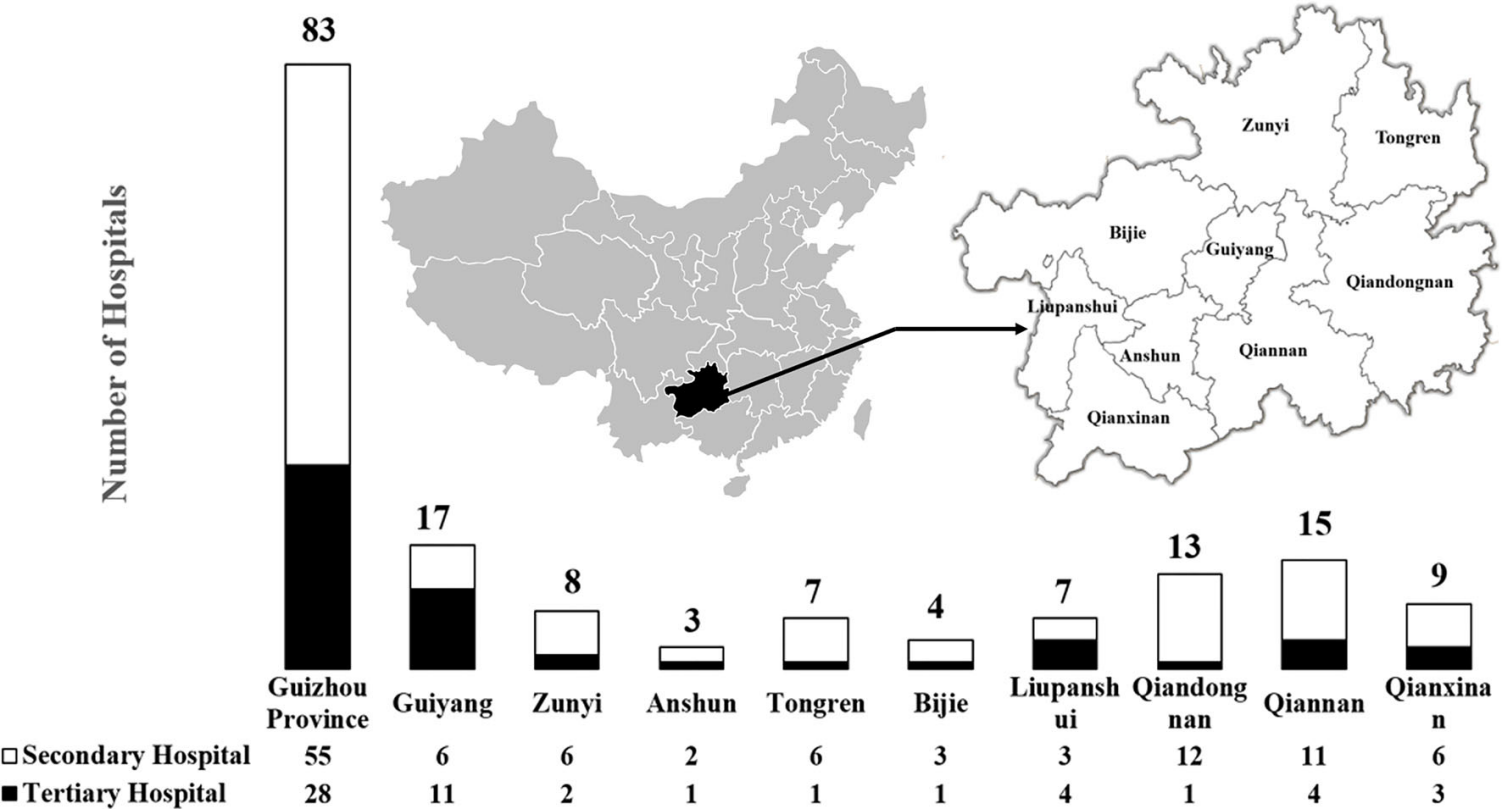
The characteristics of included hospitals.

As shown in **Figure 1**, this study included 83 hospitals (55 tertiary and 28 secondary hospitals) from nine regions (Guiyang, Zunyi, Anshun, Tongren, Bijie, Liupanshui, Qiandongnan, Qiannan, and Qianxinan) covering the entire Guizhou Province.

### The State of Clinical Pharmacist-Led Consultation for Infectious Diseases in Guizhou Province

As shown in **Table 1**, CPS and CPC for ID were developed in 79 (91.86%) and 50 (60.24%) of 83 hospitals, respectively. The development of CPS and CPC for ID in the tertiary hospitals was better than that in the secondary hospitals. The proportion of hospitals with CPS (75.00%) or CPC (25.00%) for ID in Bijie region was the lowest among the nine regions.

**Table 1:** The situation of clinical pharmacy service and clinical pharmacist in hospital in Guizhou Province.

Total/subgroup		N of Hospitals with CPS (%)	N of hospitals with CPC for ID (%)	N of hospitals with CP (%)	N of hospitals with enough CP (%)	N of hospitals with ID, respiratory, or general CP (%)
**Guizhou Province**		79 (91.86)	50 (60.24)	62 (74.70)	18 (21.69)	55 (66.26)
**Rank**	**Secondary hospital**	51 (89.47)	27 (49.09)	37 (67.27)	6 (10.91)	31 (56.36)
	**Tertiary hospital**	28 (96.55)	23 (82.14)	25 (89.29)	12 (42.86)	24 (85.71)
**Region**	**Guiyang**	16 (94.12)	14 (82.35)	14 (82.35)	5 (29.41)	13 (76.47)
	**Zunyi**	8 (100.00)	6 (75.00)	8 (100.00)	4 (50.00)	8 (100.00)
	**Anshun**	3 (100.00)	2 (66.67)	3 (100.00)	1 (33.33)	3 (100.00)
	**Tongren**	7 (100.00)	4 (57.14)	5 (71.43)	2 (28.57)	5 (71.43)
	**Bijie**	3 (75.00)	1 (25.00)	3 (75.00)	1 (25.00)	2 (50.00)
	**Liupanshui**	6 (85.71)	4 (57.14)	5 (71.43)	1 (14.29)	4 (57.14)
	**Qiandongnan**	12 (92.31)	7 (53.85)	9 (69.23)	0 (0.00)	8 (61.54)
	**Qiannan**	15 (100.00)	7 (46.67)	9 (60.00)	2 (13.33)	7 (46.67)
	**Qianxinan**	9 (100.00)	5 (55.56)	6 (66.67)	2 (22.22)	5 (55.56)

N, number; CPS, clinical pharmacy service; CPC, clinical pharmacist-led consultation; ID, infectious diseases; CP, clinical pharmacist.

### The Situation of Clinical Pharmacist in Guizhou Province

**Figure 2:**
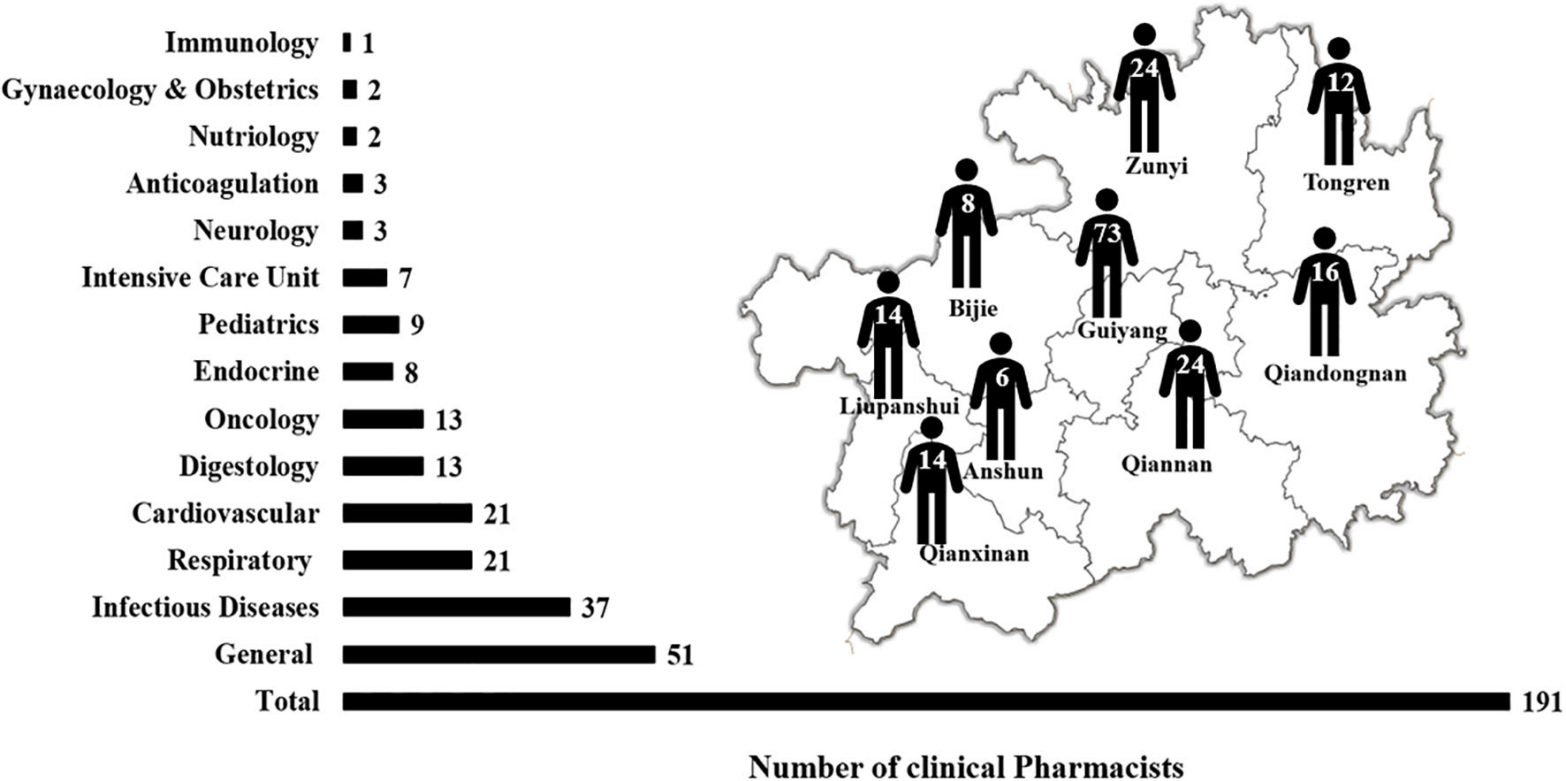
The major and distribution of clinical pharmacists.

**Table 1** indicates that 62 hospitals (74.70%) had clinical pharmacists, but only 18 of them (21.69%) had enough clinical pharmacists (≥3 in secondary hospitals or ≥5 in tertiary hospitals) according to the requirement of Chinese Hospital Classification Management Standard. The proportion of hospitals with clinical pharmacists or with enough clinical pharmacists was lower in secondary hospitals than those in tertiary hospitals. A total of 191 clinical pharmacists with 14 different backgrounds were unevenly distributed in Guizhou Province (**Figure 2**). The majority (57.07%) of clinical pharmacists specialized in ID, respiratory, and general diseases, but only 55 hospitals (66.26%) were equipped with such clinical pharmacists. The proportion of hospitals with ID, respiratory, or general clinical pharmacist was also lower in secondary hospitals (56.36%) compared to that in tertiary hospitals (85.71%).

### Factors Associated With Implementation of Clinical Pharmacist-Led Consultation for Infectious Diseases

The implementation of CPC for ID was associated with several factors. As shown in **Table 2**, the hospital rank, origin, and staffing enough clinical pharmacists did not significantly affect the enforcement of CPC for ID (*P* > 0.05). However, the *P* value (0.059) of staffing enough clinical pharmacists was close to the threshold (0.05), which implied that this factor might be a potential positive factor for implementation of CPC for ID (*AOR* =14.339, 95% *CI*: 0.903 to 227.778). When other factors were controlled, staffing clinical pharmacists with specialty training in ID, respiratory, or general diseases significantly increases the possibility of developing CPC for ID (*AOR* = 7.298, 95% *CI*: 2.232 to 23.858, *P* = 0.001).

**Table 2:** The results of Logistic regression model (N = 83).

Variable		*P* value	Adjusted odds ratios	95% Confidence interval
**Hospital rank: tertiary *vs*. secondary hospital**		0.593	1.439	[0.379, 5.465]
**Hospital region:**	**Zunyi *vs*. Guiyang**	0.135	0.172	[0.017, 1.734]
	**Anshun *vs*. Guiyang**	0.170	0.148	[0.096, 2.270]
	**Tongren *vs*. Guiyang**	0.253	0.248	[0.023, 2.715]
	**Bijie *vs*. Guiyang**	0.121	0.043	[0.001, 2.289]
	**Liupanshui *vs*. Guiyang**	0.432	0.410	[0.044, 3.791]
	**Qiandongnan *vs*. Guiyang**	0.393	0.438	[0.066, 2.905]
	**Qiannan *vs*. Guiyang**	0.263	0.344	[0.053, 2.230]
	**Qianxinan *vs*. Guiyang**	0.364	0.383	[0.048, 3.048]
**Enough clinical pharmacist: yes *vs*. no**		0.059	14.339	[0.903, 227.778]
**ID, respiratory, general clinical pharmacist** **^a^**: **yes *vs*. no**		0.001	7.298	[2.232, 23.858]
**Constant**		0.714	0.750	[0.161, 3.494]

a P < 0.05.

### Acceptance Rate of Consultation Suggestion

**Figure 3:**
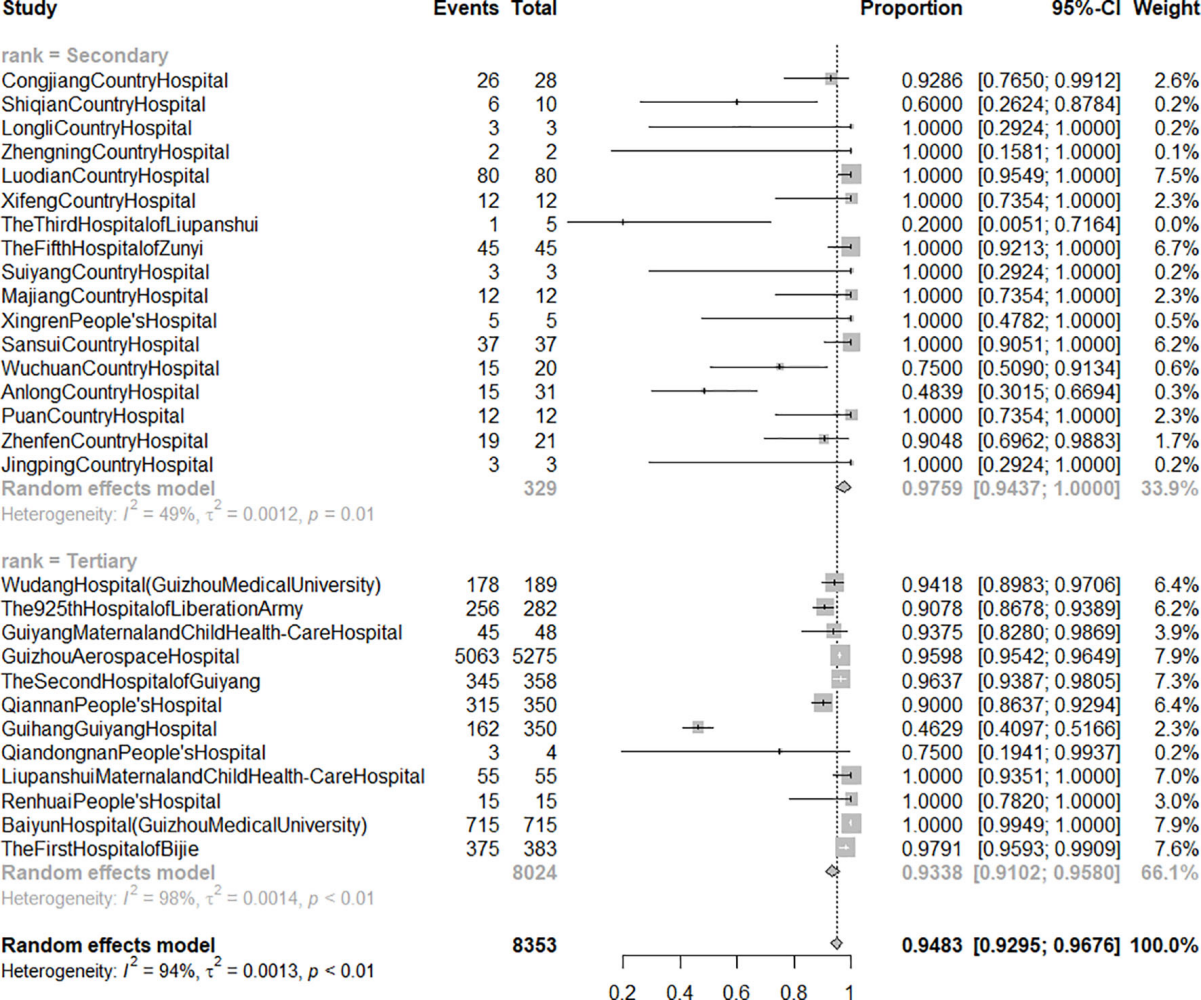
The forest graph of acceptance rate of consultation suggestion from clinical pharmacist by hospital rank. Secondary: secondary hospital; tertiary: tertiary hospital. Events: number of patients whose treatment followed pharmacists' suggestions. Total: number of patients who applied for clinical pharmacist-led consultation for infectious diseases.

A total of 29 hospitals (34.94%) including 8,353 patients reported the ARCS. The raw data had a non-normal distribution and were thus log-transformed prior to analysis. **Figure 3** shows that the ARCS ranged from 46.29 to 100.00% with a pooled rate of 94.83% (95% *CI*: 92.95 to 96.76%), and the heterogeneity among ARCS reported by different hospitals was significant (*I^2^* = 94%). Subgroup analyses suggested that hospital rank was a source of heterogeneity. The pooled ARCS of secondary hospitals was 97.59% (95% *CI*: 94.37 to 100.00%, *I^2^ = *49%), while the ARCS of tertiary hospitals ranged from 46.29 to 100.00% and the pooled ARCS could not be calculated using meta-analysis method due to significant heterogeneity (*I^2^* = 98%).

### Effective Response Rate After Acceptance of Pharmacists' Suggestions

**Figure 4:**
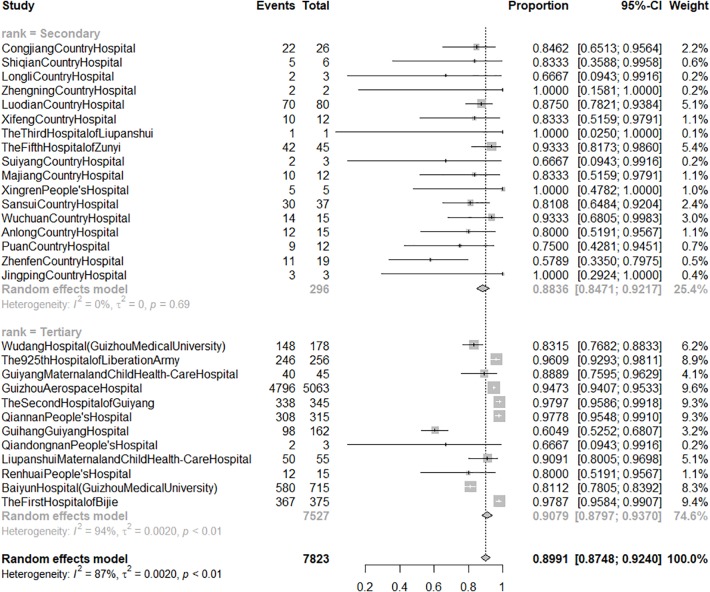
The forest graph of effective response rate of patients treated after acceptance of pharmacists' suggestions by hospital rank. Secondary: secondary hospital; tertiary: tertiary hospital. Events: number of responsive patients whose treatment followed pharmacists' suggestions. Total: number of patients whose treatment followed pharmacists' suggestions.

A total of 29 hospitals (34.94%) involving 7,823 patients reported the ERRAPS. The raw data had a non-normal distribution and were thus log-transformed before analysis. ERRAPS ranged from 57.89 to 100.00% with a pooled rate of 89.91% (95% *CI*: 87.48 to 92.17%), and the heterogeneity among different hospitals was significant (*I^2^* = 87%). Subgroup analyses (**Figure 4**) indicated hospital rank as a source of heterogeneity. The pooled ERRAPS of secondary hospitals was 88.36% (95% *CI*: 84.71 to 92.17%, *I^2^* = 0%), while the ERRAPS of tertiary hospitals ranged from 60.49 to 97.97% and the pooled ERRAPS could not be calculated using a meta-analysis method because of significant heterogeneity (*I^2^* = 94%).

**Figure 5:**
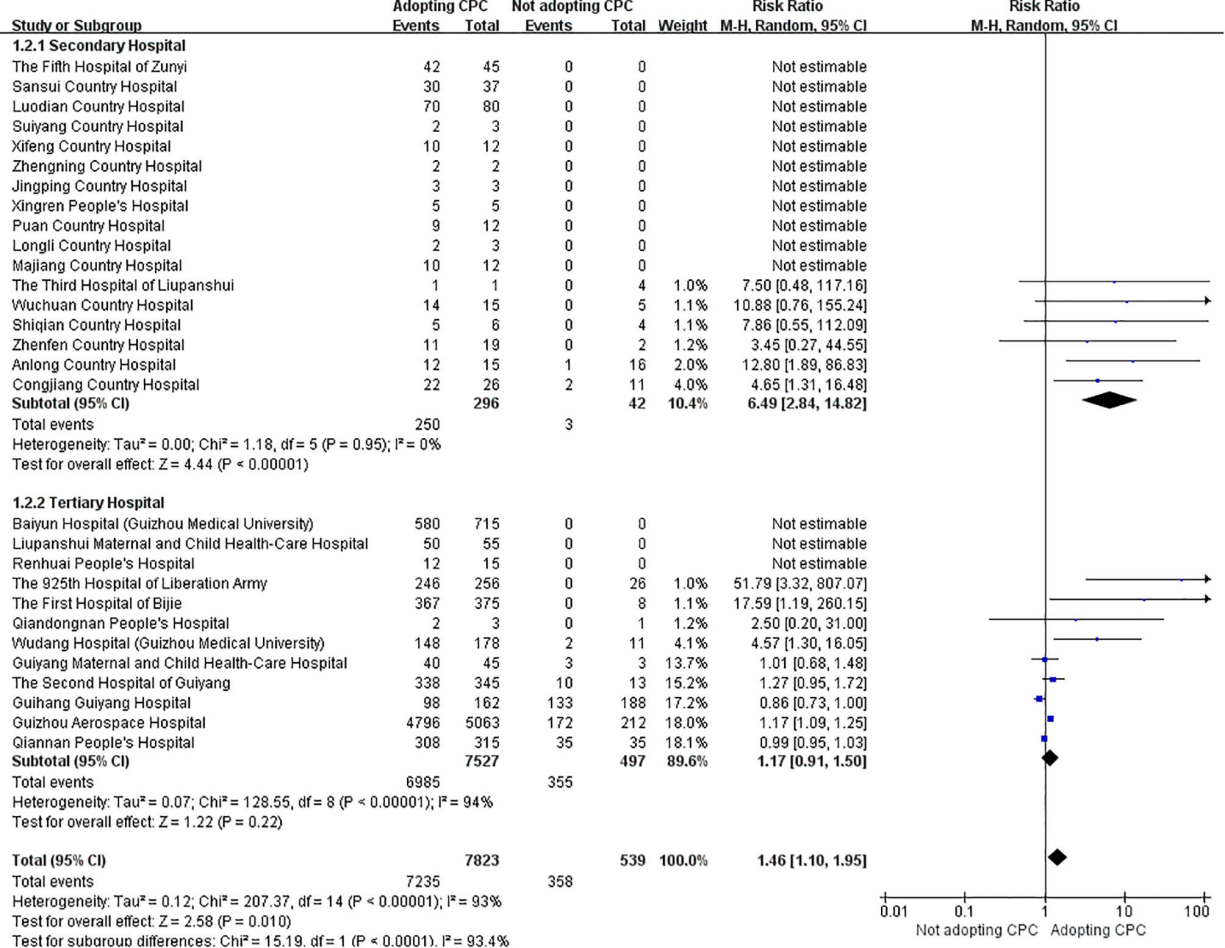
Meta-analysis forest plot of effective response rate of patients in two group. CPC, clinical pharmacists' consultation opinion.

To compare the effective response rate between patients whose treatment followed the pharmacists' suggestions and those whose treatment did not, a pairwise meta-analysis using *RR* with 95% *CI* was conducted with a random-effect model. The heterogeneity among different hospitals was significant (*I^2^* = 93%) and subgroup analyses identified hospital rank as a source of heterogeneity (**Figure 5**). For the secondary hospital subgroup, the pooled *RR* was 6.49 (95% *CI*: 2.84 to 14.82, *I^2^* = 0%), meaning that taking pharmacists' suggestions in secondary hospitals could significantly improve the prognosis of patients with ID. But for the tertiary hospital subgroup, the pooled *RR* could not be calculated using random-effect model due to significant heterogeneity (*I^2^* = 94%). Four hospitals (The 925^th^ Hospital of Liberation Army, the First Hospital of Bijie, Wudang Hospital affiliated with Guizhou Medical University, and Guizhou Aerospace Hospital) which included 6,129 patients reported that the prognosis of patients whose treatment utilized suggestions from clinical pharmacists was significantly improved; however, the other five hospitals (Qiandongnan People's Hospital, Guiyang Maternal and Child Health-Care Hospital, The second Hospital of Guiyang, Guihang Guiyang Hospital, and Qiannan People's Hospital) which included 1,110 patients reported that adopting suggestions of clinical pharmacists did not significantly influence the effective response rate.

## Discussion

The current survey involved 83 hospitals (55 secondary and 28 tertiary hospitals) covering 9 regions in Guizhou Province to investigate the situation of CPC for ID. We found that: 1) most hospitals have promoted CPS but the development of CPC for ID was still at an early stage; 2) although many hospitals had staffed clinical pharmacists, the number of clinical pharmacists was still inadequate; 3) ID, respiratory, and general clinical pharmacists had the highest staffing ratios; however, only a few hospitals were staffed, which limited the implementation of CPC for ID; 4) only a few hospitals developing CPC for ID followed the outcome of patients who applied for CPC; and 5) CPC intervention could improve the prognosis of ID patients in secondary hospitals (*RR* = 6.49, 95% *CI*: 2.84 to 14.82).

Although the development of CPC for ID in Guizhou is still at an early stage, the proportion of hospitals with CPC for ID in Guizhou Province increased from 50.72% (2016) (Zhang et al., 2018) to 60.24% (2018), indicating that the measures adopted by policy-makers is effective. In 2018, the number of clinical pharmacists (191) was nearly 1.78 times greater than in 2016 (107) (Zhang et al., 2018), but insufficient number of clinical pharmacists is still a critical problem in most hospitals. This study indicated that staffing ID, respiratory, or general clinical pharmacists in hospitals had a significantly positive impact on the implementation of CPC for ID, and the number of these clinical pharmacists in 2018 increased greatly compared to 2016 (109 *vs*. 56) (Zhang et al., 2018). Nevertheless, the presence of clinical pharmacists in hospitals (66.26%) is still limited, which restricts the development of CPC for ID in Guizhou. Training qualified ID clinical pharmacists for more grassroots medical institutions is believed to be the most important task to improve CPC for ID.

The proportion of hospitals reporting complete patient outcomes increased greatly from 20.29% (Zhang et al., 2018) to 58% from 2016 to 2018, indicating that more hospitals are beginning to pay attention to patient follow-ups. The pooled ARCS for secondary hospitals in Guizhou (97.59%, 95% *CI*: 94.37 to 100.00%) was slightly higher than that in the whole country (93.97%, 95% *CI*: 89.43 to 96.64%) (Zhang et al., 2019a), showing that, for the secondary hospitals, clinicians in the province with limited medical resource were more likely to collaborate with the clinical pharmacists and adopt their suggestions. Furthermore, the excellent ERRAPS for secondary hospitals in Guizhou (88.36%, 95% *CI*: 84.71 to 92.17%) also indicated the prominent effect of CPC on ID patients' prognosis. To further evaluate the effectiveness of CPC, the patients whose treatment followed the suggestions of clinical pharmacists were compared to those whose treatment did not follow the suggestions (the control group). The results of meta-analysis demonstrated that, for the secondary hospitals in Guizhou, CPC intervention could significantly improve the prognosis of ID patients (*RR* = 31.28, 95% *CI*: 10.15 to 96.36), which is consistent with the results of a previous systematic review (*RR* = 2.08, 95% *CI*: 1.41 to 3.06) (Zhang et al., 2019a) and a single center cohort study (*OR* = 1.738, 95% *CI*: 1.028 to 2.940) (Zhang et al., 2019b). In fact, clinical pharmacists can participate in the treatment of ID through a variety of ways (e.g., round with clinicians, discuss with patients, connect with microbiologists and nurses, develop a therapeutic scheme, adjust the dosage regimen according to pharmacokinetic-pharmacodynamic results, and monitor adverse events) during the consultation process (Zhang et al., 2019a). The multiple contributions of clinical pharmacists can help clinicians to optimize the treatments and improve patient outcomes. However, the effectiveness of CPC in the tertiary hospitals was controversial, which can be attributed to the influence of uncontrolled confounding factors (e.g., medical comorbidities, severity of infections, and liver function of patients) (Zhang et al., 2019b).

We found that hospital rank was a source of heterogeneity, which could be attributed to the difference between secondary and tertiary hospitals on medical resources and severity of ID patients. Due to the insufficient diagnostic and therapeutic capabilities in secondary hospitals, the clinicians prefer to ask for pharmacists' opinions. Moreover, patients with serious ID and resistant bacterial infections in secondary hospitals were usually transferred to adjacent tertiary hospitals. Therefore, the prognosis of patients and the effectiveness of CPC intervention in secondary hospitals were much better than those in tertiary hospitals.

The limitations of this study must be acknowledged. Firstly, although we conducted the meta-analysis using a random-effect model and performed subgroup analyses, the heterogeneity among different tertiary hospitals was still significant, which implies the presence of other unknown factors causing the heterogeneity. Due to the limited information in this survey, the complete causes of heterogeneity could not be examined. Secondly, due to the absence of individual patient data, we conducted the analyses on a hospital level and could not control the confounding factors. Nevertheless, this survey enables us to understand the baseline situation about CPC in Guizhou Province and urges us to initiate a multicenter, prospective cohort study based on a regional registry network platform to further evaluate the effectiveness of CPC in the treatment of ID.

Recently, increasing evidence has corroborated the positive impact of clinical pharmacists on rational use of antimicrobial agents (Brink et al., 2017; Li et al., 2017; Kulwicki et al., 2018; Ohashi et al., 2018; Rehman et al., 2018). For instance, clinical pharmacists could help to reduce antimicrobial agent usage and costs of hospitalization (Shen et al., 2011; Magedanz et al., 2012; Waters, 2015; Zhou et al., 2015; Brink et al., 2016; Li et al., 2017; Zhang et al., 2017; Rehman et al., 2018), shorten the duration of antimicrobial treatment and length of hospital stay (Dunn et al., 2011; Grill et al., 2011; Shen et al., 2011; Apisarnthanarak et al., 2015; Ellis et al., 2016; Li et al., 2017), as well as improve life quality and hospital mortality (Li et al., 2017; Ohashi et al., 2018; Rehman et al., 2018). But insufficient number of clinical pharmacist staff and backward development of CPS are still common in Chinese grassroots medical institutions. For these institutions, CPC may be a more appropriate CPS intervention in the treatment of ID, which can be shared with other developing countries or regions confronting the similar situation (e.g., Sub-Saharan Africa and Asia).

## Conclusion

As a survey conducted in a less developed province in Western China, the study reflects the current situation regarding the development of CPS in economically disadvantaged areas. For the grassroots medical institutions in these areas, CPC will be a promising CPS intervention, and ID field will be the breakthrough point for CPS. The policy/decision-makers should be cognizant of the critical value of CPC within grassroots healthcare systems. Considering the limitations of this survey, we are planning to conduct a registry study in across all of Guizhou province to further evaluate the effectiveness of CPC for ID from a regional perspective.

## Data Availability Statement

The raw data supporting the conclusions of this article will be made available by the authors, without undue reservation, to any qualified researcher.

## Ethics Statement

The studies involving human participants were reviewed and approved by the Ethics Committee of Guizhou Provincial People's Hospital. Written informed consent for participation was not required for this study in accordance with the national legislation and the institutional requirements.

## Author Contributions

JZ, WZ, RH, JX, XQ, SX, and QC collected the data. JZ and CX involved in statistical analysis. JZ, CX, WZ, and RH drafted the manuscript. JZ, CX, WZ, RH, JX, XQ, SX, and QC interpreted the data. All authors formulated and designed the study, performed critical revision of the manuscript, and approved a final version of the manuscript to be published including the authorship list.

## Funding

This work was supported by the Medical Science Research Program of Beijing Health Care Foundation (YWJKJJHKYJJ-B17444).

## Conflict of Interest

The authors declare that the research was conducted in the absence of any commercial or financial relationships that can be construed as a potential conflict of interest.
