# Supplementary material for: The Clinical Pharmacist-Led Consultation for Infectious Diseases in Guizhou Province, China: A Survey Among Hospital Pharmacies
Source: Front Pharmacol. 2020 Feb 27;11:149. doi: 10.3389/fphar.2020.00149 (PMC7056738; doi:10.3389/fphar.2020.00149)
Supplement: Supplementary file 1 [file DataSheet_1.docx]

**Table S1. Ethical Approval Documentation (Chinese)**


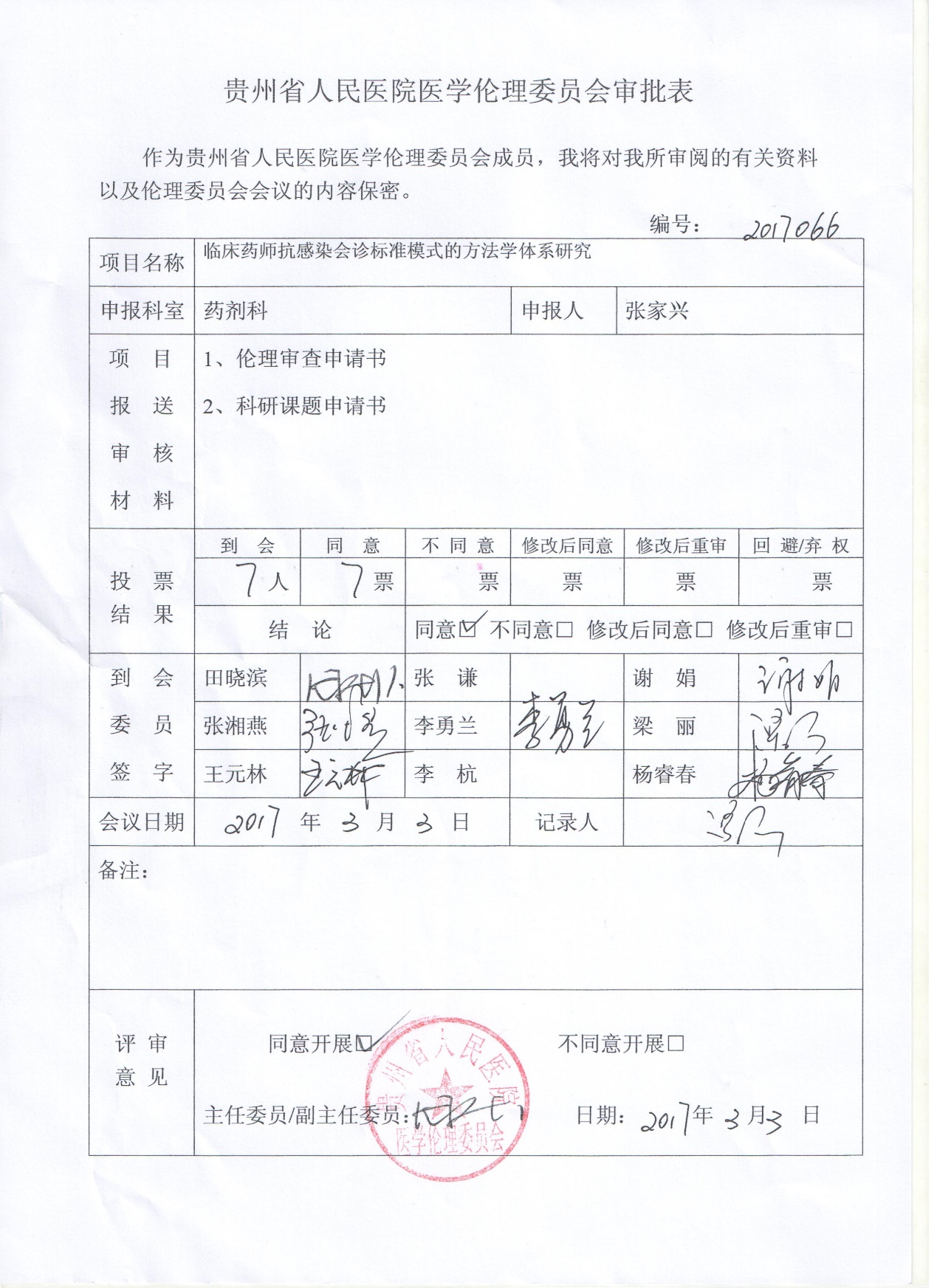


**Table S2. Ethical Approval Documentation (Translation to English)**

**Approval form of the Medical Ethics Committee of Guizhou Provincial People’s Hospital**

As a member of the Medical Ethics Committee of Guizhou Provincial People’s Hospital, I will keep secret for the materials which I reviewed and the content of the conference.

The Number: 2017066

| Title of the project | Methodological research on the standard procedure of clinical pharmacists’ consultation for infectious diseases. | | | | | |
| --- | --- | --- | --- | --- | --- | --- |
| Department | Department of Pharmacy | | | Applicant | Zhang Jiaxing | |
| The materials for ethical review | 1. Application for ethical review  2. Application for the study projects | | | | | |
| The results of voting | Attendance  (n) | Agree  (n) | Disagree  (n) | Agree after the project was revised  (n) | Review after the project was revised  (n) | Avoidance / abstention  (n) |
|  | 7 | 7 |  |  |  |  |
|  | Conclusion | | Agree√ Disagree□  Agree after the project was revised□  Review after the project was revised□ | | | |
| Member of the ethics committee | Tian Xiaobing |  | Zhang Qian |  | Xie juan |  |
|  | Zhang Xiangyan |  | Li Yonglan |  | Liang Li |  |
|  | Wang Yuanlin |  | Li Hang |  | Yang Ruichun |  |
| Conference date | 3/3/2017 | | | Conference recorder | Wang Han | |
| Remark: None | | | | | | |
| Review comments | Agree to conduct this project√  Disagree to conduct this project□  Chairman: Date: 3/3/2017 | | | | | |
